# Cardiorespiratory Fitness and the Risk of All-Cause, Cardiovascular and Cancer Mortality in Men with Hypercholesterolemia

**DOI:** 10.3390/jcm11175211

**Published:** 2022-09-03

**Authors:** Xuemei Sui, Mark A. Sarzynski, Nicole Gribben, Jiajia Zhang, Carl J. Lavie

**Affiliations:** 1 Department of Exercise Science, Arnold School of Public Health, University of South Carolina, Columbia, SC 29208, USA; 2 Department of Epidemiology and Biostatistics, Arnold School of Public Health, University of South Carolina, Columbia, SC 29208, USA; 3 Department of Cardiovascular Diseases, John Ochsner Heart and Vascular Institute, Ochsner Clinical School, University of Queensland School of Medicine, New Orleans, LA 70121, USA

**Keywords:** cardiorespiratory fitness, hypercholesterolemia, mortality

## Abstract

**Background:** Whether higher cardiorespiratory fitness (CRF) confers protection against cardiovascular disease (CVD) in individuals with manifest hypercholesterolemia is poorly understood. **Methods:** Participants were 8920 men aged 20–82 years with hypercholesterolemia but no history of CVD and/or cancer and who received a preventive examination at the Cooper Clinic in Dallas, TX, USA, during 1974–2001. CRF was quantified as maximal treadmill test duration and was grouped for analysis as low, moderate, or high based on the traditional Aerobics Center Longitudinal Study cutpoints. Using Cox regression analyses, we computed hazard ratios and 95% confidence intervals for risk of mortality based on CRF. **Results:** During an average of 17 years of follow-up, 329 CVD and 290 cancer deaths occurred. After control for baseline age, examination year, body mass index, total cholesterol, smoking, alcohol intake, physical activity, hypertension, diabetes, and parental history of CVD, hazard ratios (95% confidence interval) for CVD deaths across moderate and high categories of CRF (with low fit as referent) were: 0.66 (0.50–0.87) and 0.55 (0.39–0.79), respectively. There was an inverse association between CRF and CVD death among normal-weight (trend *p* < 0.0001), younger (<60 y, trend *p* = 0.01), and inactive men (trend *p* = 0.002). However, no significant association was found between CRF and cancer mortality. **Conclusions:** Among men with hypercholesterolemia, higher CRF was associated with a lower risk of dying from CVD independent of other clinical risk factors. Our findings underscored the importance of promoting CRF in the primary prevention of CVD in patients with hypercholesterolemia.

## 1. Introduction

Cardiovascular disease (CVD) is a growing problem and continues to be the leading cause of mortality, with 219.4 deaths per 100,000 people in the United States (US) and approximately 31.5% of all deaths in the world based on the most recent American Heart Association report from 2020 [1]. In every year since 1919, CVD accounted for more deaths than any other major cause of death in the US. Of these deaths, about 13% can be attributed to high cholesterol, one of the major CVD risk factors [2]. In 2015–2018, nearly 12% of US adults aged 20 and older had total cholesterol higher than 240 mg/dL and nearly 94 million (38%) had levels higher than 200 mg/dL [1].

Leisure-time physical activity (PA) [3,4,5,6] and cardiorespiratory fitness (CRF) [7,8,9,10,11] are inversely associated with CVD morbidity and mortality. Our earlier studies also linked higher CRF to lower mortality risk from CVD [7,12,13] and we found that compared to low CRF, moderate and high CRF had about a 20% and 40% lower risk of CVD mortality, respectively. The cardioprotective effect of PA and aerobic exercise has been partially attributed to favorable changes in cholesterol and triglyceride levels [14,15,16,17]. Higher PA and CRF are associated with a lower risk of developing hypercholesterolemia [18,19,20,21,22]. In addition, accumulating evidence shows that aerobic exercise can lower blood cholesterol in both normolipidemic and hyperlipidemic subjects [23,24]. The National Cholesterol Education Program (NCEP) therefore recommended that hypercholesterolemic patients increase their PA for primary prevention of heart disease [25]. PA and CRF have been considered as two different risk factors, as PA is a behavior while CRF is an attribute. However, the primary determinant of CRF is activity behavior, although genetics makes a significant contribution [26,27].

In 2022, 1.9 million new cancer cases were diagnosed in the US and 609,360 cancer deaths occurred [28]. About one-fifth of all cancer cases are estimated to be attributable to insufficient PA [29]. Most previous studies on associations between PA and cancer were based on self-reported PA measurement, which is prone to misclassification [30], while a few studies were based on CRF assessment [7,31,32,33,34,35,36,37,38]. A majority of these studies, including our earlier studies [7,31], showed an inverse association between CRF and cancer mortality.

Whether higher CRF confers protection in individuals with manifest hypercholesterolemia is poorly understood. Only one previous study examined a group of veterans with dyslipidemia and found that increased peak metabolic equivalents (METs) achieved during an exercise endurance test were independently associated with lower mortality [39]. Moreover, the combination of statin treatment and increased fitness resulted in a substantially lower mortality risk than either alone [39], reinforcing the importance of habitual PA for individuals with dyslipidemia. Therefore, the objective of this study was to extend the existing literature by examining the relationship between CRF and mortality from all causes, CVD, and cancer in men with high cholesterol (the number of deaths in women is small) to test the hypothesis that a higher level of CRF protects a population with hypercholesterolemia from dying.

## 2. Materials and Methods

### 2.1. Study Population

The participants were 10,912 men with hypercholesterolemia at baseline and who were enrolled in the Aerobics Center Longitudinal Study (ACLS). As described in previous studies [12,40], these men all received a preventive examination at the Cooper Clinic in Dallas, Texas, USA, from 1974–2001. Participants came to the clinic for periodic preventive health examinations and for counseling regarding diet, exercise, and other lifestyle factors associated with an increased risk of chronic disease. Many were sent by their employers for the examination, some were referred by their doctors, and others self-referred. The participants were thus volunteers. Informed consent was obtained from each participant before beginning the study. The study was reviewed and approved by the Institutional Review Board at the Cooper Institute. Participants were excluded from the present study if at baseline they did not achieve at least 85% of aged-predicted maximal heart rate (220-age) during the treadmill test (*n* = 192); had an abnormal resting or exercise electrocardiogram (*n* = 815); reported a history of myocardial infarction (*n* = 203), stroke (*n* = 23), or cancer (*n* = 223); or were underweight (BMI < 18.5 kg/m^2^) (*n* = 536). These criteria resulted in 8920 men with high cholesterol aged 20–82 years who were followed up from the date of their baseline examination until their date of death or 31 December 2003. Only 26 deaths from CVD and 47 deaths from cancer were available among women. Due to the insufficient statistical power for a meaningful analysis, we excluded women from the current study.

### 2.2. Clinical Examination

The clinical examination took place at the Cooper Clinic and was carried out by a clinic physician. Antecubital venipuncture was used to obtain blood samples. An extensive questionnaire was also completed by participants with information regarding demographic characteristics, health history, family medical history, and a health habit inventory. Blood chemistry analyses were performed in the laboratory at the Cooper Clinic using standard bioassays. The Cooper Clinic participates in and meets all quality control standards set forth by the Centers for Disease Control Lipid Standardization Program. Hypercholesterolemia was defined as fasting blood cholesterol levels of ≥240 mg/dL (6.20 mmol/L) [41] at baseline. Blood pressure was taken with auscultatory methods following the American Heart Association protocol [42]. Men who reported a previous physician diagnosis of hypertension were classified as hypertensive. Body mass index (BMI) was calculated and men were assigned to categories of normal weight (BMI 18.5–24.9 kg/m^2^) and overweight or obese (BMI ≥ 25.0 kg/m^2^).

Smoking and alcohol intake habits were assessed using a questionnaire. Smoking habits were categorized into current smoker or not (including former smoker and never smoker). Drinks per week were computed with one drink standardized to 12 ounces of beer (3.41 dL), 5 ounces of wine (1.421 dL), or 1.5 ounces of liquor (0.4262 dL). Heavy alcohol intake was defined as >14 drinks per week in men [43]. Physical inactivity was defined by self-report as having not engaged in leisure-time PA in the 3 months prior to the examination [44]. The presence of individual or parental CVD or cancer was assessed on a standardized health history questionnaire.

### 2.3. Cardiorespiratory Fitness

CRF was quantified with the use of a treadmill test using a modified Balke protocol [12,45]. Participants began walking on a treadmill (CMT 22-63, Langley, WA, USA) at a speed of 3.3 miles per hour/88 m per minute at a 0% incline. After one minute, the incline was increased to 2% and increased 1% every minute thereafter. Speed was maintained until 25 min had passed, after which the speed was increased by 0.2 miles per hour/5.4 m per minute until the test was terminated. The test was terminated when the participant was exhausted and chose to self-terminate or if medical reasons dictated that a physician order the test to be terminated. Participants were then grouped based on the duration that they were able to continue the test. The total duration of this test in men has been shown to correlate highly with measured maximum oxygen uptake (r = 0.92) [46]. The participants were classified into low- (the lowest 20%), moderate- (the middle 40%), and high-fitness groups (the upper 40%) [40]. These groups were respective to the age- and sex-specific distributions of maximum exercise duration throughout the overall ACLS cohort. The purpose of this classification was to maintain consistency throughout our methods due to the lack of a widely accepted clinical categorization of CRF.

### 2.4. Mortality Surveillance

Mortality surveillance was conducted by monitoring the National Death Index. Death certificates were collected from states in which participants’ deaths occurred and cause of death was then ascertained from the certificate. The National Index has a similar accuracy to the mortality data determined by the Endpoints Review Committee, which reviews death certificates and medical records relevant to an event and study to make their decision [47]. For the National Death Index, the sensitivity for CVD mortality is 90% and the specificity is 93%. Before 1999, International Classification of Diseases, Ninth Revision (ICD-9) codes were used to identify CVD (390–449.9) and cancer (140–208) mortality, while Tenth Revision (ICD-10) codes were used from 1999–2003 (I00-I78 for CVD and C00-C97 for cancer) [48].

### 2.5. Statistical Analysis

Baseline characteristics of the population were examined according to CRF categories. Trends in baseline characteristics by CRF were tested using F tests. Follow-up time among survivors was calculated as the difference between the date of the baseline examination date and 31 December 2003. Follow-up time among decedents was computed as the difference between the baseline examination date and the reported date of death. Cox regression analysis was used to estimate hazards ratios (HRs) and 95% confidence intervals (CIs) of all-cause, CVD, and cancer deaths according to CRF categories. The proportional hazards assumption was examined by comparing the cumulative hazards plots across CRF groups; no appreciable violations were noted. Three models were tested: Model 1 adjusted for baseline age and examination year; Model 2 adjusted for variables in Model 1 plus baseline total cholesterol (mg/dL), current smoking (yes or no), heavy drinking (yes or no), physically inactive (yes or no), and parental history of CVD (when all-cause or CVD mortality as the outcome) or cancer (when cancer mortality was the outcome) (present or not); and Model 3 adjusted for all variables in Model 2 plus BMI (kg/m^2^) and presence or absence of hypertension or diabetes. Tests of linear trends were computed using ordinal scoring. Finally, we performed stratified analyses according to age (<60 vs. ≥60), weight status (BMI < 25 vs. ≥25), and PA (inactive vs. active) categories to test the effect modification from these three factors. All *p*-values were two-sided and *p* < 0.05 was regarded as statistically significant.

## 3. Results

There was a total of 795 deaths (329 deaths due to CVD and 260 deaths due to cancer) during the average of 17 years of follow-up. The mean baseline age for participants was 45.9 (8.6) years. Compared to individuals classified as having high CRF, those with low CRF were younger, more likely to be smokers and physically inactive, and had better glucose and lipid profiles, but reported more heavy alcohol intake (Table 1).

Rates of all-cause, CVD, and cancer mortality are shown in Table 2. An inverse gradient of all-cause mortality was observed across incremental CRF groups (Model 1, *p for linear trend* < 0.0001). After adjustment for covariates (Model 2), men with moderate and high CRF had a 40% and 45% lower mortality risk than men with low CRF (*p for linear trend* < 0.0001). The inverse association remained significant after additional adjustment for BMI, hypertension, and diabetes (Model 3, *p for linear trend* = 0.002). A similar inverse pattern of association was observed between CRF and CVD mortality; however, no significant association was found between CRF and cancer mortality.

**Figure 1:**
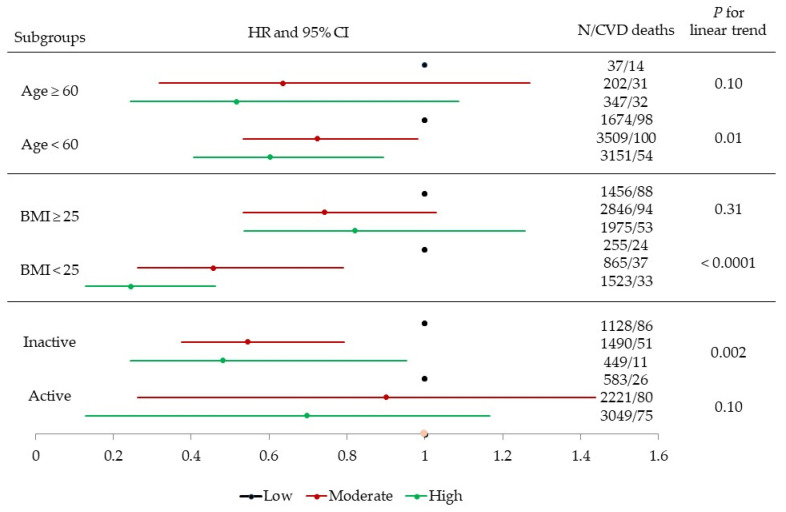
Multivariable-adjusted hazard ratios (HRs) and 95% confidence intervals (CIs) of cardiovascular disease (CVD) mortality across fitness (low, moderate, and high) and age (<60 and ≥60), body mass index (BMI, 18.5–25 and ≥25), and physical activity (inactive and active) groups. Adjusted according to baseline age, examination year, total cholesterol, current smoking (yes or no), heavy alcohol intake (yes or no), physically inactive (yes or no), BMI (kg/m^2^), parental history of CVD, and presence or absence of hypertension or diabetes.

We also examined whether baseline age, BMI, and self-reported PA (Figure 1) modified the association between CRF and CVD mortality. After adjusting for all potential covariates, there was a significant inverse gradient of CVD death risk across incremental CRF levels among younger men (age < 60) (*p* for linear trend = 0.01), men with normal BMI (*p* for linear trend < 0.0001), and men who were physically inactive (*p* for linear trend = 0.002), but not among older men (age ≥ 60) (*p* for linear trend = 0.10), overweight/obese men (*p* for linear trend = 0.31), and men who were physically active (*p* for linear trend = 0.10). Similar patterns of associations were observed between CRF and all-cause mortality for the effect-modification analyses (results not shown).

## 4. Discussion

The inverse gradient of all-cause and CVD mortality across CRF groups in men with hypercholesterolemia was a primary finding in this study, even after adjustment for total cholesterol, BMI, hypertension, and diabetes. This indicated an independent association between CRF and mortality from all causes and CVD among men with high cholesterol, who may benefit from an improvement in CRF. However, we did not find a statistically significant relationship between CRF and cancer mortality. The third key finding was that age, BMI, and PA modified the association between CRF and all-cause and CVD mortality. The inverse association was only observed in younger men less than 60 years old, those with a normal BMI, and those who were physically inactive. These subgroup analyses suggested that improving CRF and losing/maintaining weight earlier in life may be important since the protective associations of CRF were not observed in older or overweight/obese men.

Our findings regarding decreases in mortality due to all causes and CVD with increased CRF were consistent with earlier studies from the ACLS [7,12,13] as well as other well-known cohort studies, including but not limited to the Henry Ford Exercise Testing Project [10], Veterans Affairs Medical Center [49,50], CARDIA [51], Tokyo Gas Co. from Japan [52], and HUNT from Norway [53]. In these previous studies, cholesterol was controlled as a risk factor when assessing the relationship between CRF and mortality. The current study not only controlled total cholesterol, but also focused on a high-risk population with elevated total cholesterol. The multivariable adjusted results showed high-CRF men had 31% and 45% lower all-cause and CVD mortality, respectively, compared with low-CRF men. These findings suggested that improving CRF can override the additional risk of high cholesterol [54]. Our findings were consistent with those of the Veterans Affairs Medical Center study [39], which reported that among those not treated with statins, the all-cause mortality risk decreased as fitness increased; for highly fit individuals (>9 MET; *n* = 1498), the HR was 0.53 (95% CI 0.44–0.65; *p* < 0.0001) compared with the least-fit individuals (≤5 METs; *n* = 1024) (HR = 1.35; 95% CI 1.17–1.54; *p* < 0·0001).

Previous studies addressed both CRF and PA simultaneously in populations with mortality and clinical data [31,33,44,55,56,57]. Most of these studies adjusted one variable as a confounder while analyzing the other variable as the exposure [31,55,56,57]. Only few of them reported an association between CRF and mortality across different PA levels [33,44]. In this study, the association between CRF and mortality due to all causes and CVD was significant among men who were physically inactive but not among those who were active. Inactive participants with high levels of CRF showed a significant 52% risk reduction in CVD mortality compared to their low CRF peers; meanwhile, the risk of CVD death in active men did not differ across the low, moderate, or high CRF groups. These findings were not only consistent with our previous report [44], but also with a 2009 Korean study by Park et al. that also observed effect modification of PA on the relationship between CRF and mortality (i.e., only significant in inactive men) [33]. One explanation was the small number of deaths among inactive men with high CRF (32 deaths due to all causes and only 11 due to CVD). Nonetheless, these studies provided evidence that the relationship between CRF and all-cause/CVD mortality may be modified by PA. Based on the findings, it is even more important for those inactive individuals with high cholesterol to gain mortality benefits by increasing their PA levels. Sufficient levels of PA have been linked to a wide range of health benefits, including but not limited to physical benefits (for example, lower CVD risk factors such as high levels of cholesterol), mental health benefits (for example, lower risk of depression), and social benefits [58]. Increasing habitual PA levels is the most significant way to increase CRF. Although genetics contributes to CRF, the principal determinant of CRF is PA [59].

Limited studies have investigated the relationship between CRF and cancer mortality in general populations worldwide [7,31,32,33,34,35,36,37,38]. Most studies reported an inverse association in men [7,31,32,33,34,36,37], while a couple of studies did not observe an association between direct measurement of CRF and cancer mortality [35,38]. More recently, non-exercise estimated CRF determined using validated prediction equations has been linked to cancer mortality [60,61]; the findings were consistent with the measurement CRF. For each 1-MET increase in the non-exercise estimated CRF, risks for cancer mortality were 0.89 (0.87–0.91) in men [61]. The current study extended not only our previous ACLS studies [7,31], but also the existing literature on high-risk populations with elevated cholesterol. Although not significant, all models showed an inverse trend across low, moderate, and high CRF. Future studies with a more diverse study population of both sexes and multiple races with more deaths from cancer are warranted to provide a better understanding of this relationship.

An increase in myocardial contractility and stroke volume may be a potential mechanism to explain the relationship between CRF and mortality within the Frank Starling Law of the Heart [62], resulting in an increased stroke volume and the heart’s increased ability to deliver oxygenated blood to the peripheral vasculature and tissues, particularly allowing the heart to pump less and with less strain when at rest, as well as increased oxygen availability for the needs of the myocardial tissue. Higher fitness levels could also potentially be blunting the progression of cholesterol buildup and atherosclerosis by assisting with maintenance of endothelial function in the vasculature [63]. Maintaining elasticity of the blood vessels may be a result of increased CRF levels independent of cholesterol levels, thus allowing the protective effect of fitness against CVD to transcend hypercholesterolemia status.

Our study did not come without limitations, such as self-reported PA and the fact that ACLS has not historically provided a representative sample of the United States. The study population was limited to predominantly white, well-educated, middle- to upper-class adults. This limited the generalizability of the study’s findings, although this limitation should not affect the study’s internal validity. Although undetected subclinical diseases are always a concern in observational studies, this was less likely to have occurred in this cohort due to the comprehensive physical examination by a physician and the thorough clinical assessment completed by each participant. There was insufficient information about medication use or dietary habits to include these factors in the analysis. Limited numbers of deaths among women with high cholesterol prevented a meaningful analysis. Future studies should include such information whenever possible.

Study strengths included the use of maximal exercise testing to quantify CRF, a long follow-up, multiple mortality outcomes, and a focus on a high-risk population with elevated cholesterol.

## 5. Conclusions

The current report suggests a strong inverse association for men with hypercholesterolemia between CRF and mortality from all causes and CVD. Both moderate and high levels of CRF provided significant benefits on lowering mortality (ranging from 24% to 45% lower mortality risk). However, more research is needed to explore the link between CRF and cancer mortality in this high-risk population. Healthcare professionals should consider counseling their inactive patients with high cholesterol to become physically active or/and prescribing exercise for the purpose of increasing CRF not only to potentially reduce high cholesterol, but also to reduce the risk of all-cause and CVD-related mortality.

## Figures and Tables

**Table 1:** Baseline characteristics of study participants by CRF category among men with hypercholesterolemia *.

	Total (*n* = 8920)	Low CRF (*n* = 1711)	Moderate CRF (*n* = 3711)	High CRF (*n* = 3498)	*p* for Trend
Age (y)	45.93 ± 8.65	43.61 ± 8.06	45.47 ±8.53	47.56 ± 8.75	<0.0001
BMI (kg/m^2^)	27.01 ± 3.60	29.57 ± 4.75	27.21 ± 3.07	25.55 ±2.58	<0.0001
BMI ≥ 25 (%)	70.37	85.10	76.69	56.46	<0.0001
Current smoker (%)	19.62	34.31	21.42	10.52	<0.0001
Heavy alcohol intake (%) ^†^	9.44	7.77	9.05	10.66	0.002
Physically inactive (%) ^‡^	34.38	12.65	16.7	5.03	<0.0001
CRF (maximal METs)	11.23 ± 2.22	8.56 ± 1.05	10.60 ± 1.09	13.21 ± 1.72	<0.0001
Blood pressure (mm Hg)					
Systolic	123 ± 13	125 ± 14	122 ± 13	122± 13	<0.0001
Diastolic	83 ± 9	85 ± 10	83 ± 10	82 ± 9	<0.0001
Hypertension (%) ^§^	36.24	9.27	15.25	11.73	<0.0001
Fasting glucose (mg/dL)	104.38 ± 42.27	106.61 ± 25.95	102.37 ± 18.62	105.41 ± 25.67	0.51
Diabetes mellitus (%) ^¶^	7.06	2.29	2.95	1.83	<0.0001
Total cholesterol (mg/dL)	265.15 ± 48.01	268.19 ± 29.20	265.46 ± 26.03	263.34 ± 68.82	0.003
Parental history of cancer (%)	0.92	0.15	0.37	0.40	0.61
Parental history of premature CVD (%)	32.54	6.32	13.24	12.98	0.47

Abbreviations: BMI, body mass index; CRF, cardiorespiratory fitness; SD, standard deviation; CVD, cardiovascular disease; METs, metabolic equivalents. Values shown as means ± SD. To convert the values for fasting glucose to mmol/L, multiply by 0.0555; to convert total cholesterol values to mmol/L, multiply by 0.0259. * Defined as total cholesterol ≥ 240 mg/dL (6.20 mmol/L). ^†^ Defined as alcohol drinks > 14 per week for men. ^‡^ No leisure-time physical activity during the past three months. ^§^ Defined as systolic or diastolic blood pressure ≥ 140/90 mmHg or history of physician diagnosis. ^¶^ Defined as a fasting plasma glucose concentration 126 mg/dL (≥7.0 mmol/L), a history of physician diagnosis, or insulin use.

**Table 2:** Rates and hazard ratios for mortality by CRF groups in men with hypercholesterolemia.

Outcomes			Model 1 ^†^		Model 2 ^‡^		Model 3 ^§^	
CRF Status	Deaths	Rate *	HR	95% CI	HR	95% CI	HR	95% CI
**All-cause**								
Low CRF	248	91.2	1.00	Referent	1.00	Referent	1.00	Referent
Moderate CRF	302	48.4	0.57	0.48–0.68	0.6	0.50–0.72	0.68	0.57–0.82
High CRF	245	34.8	0.51	0.42–0.61	0.55	0.44–0.68	0.69	0.55–0.87
*p* linear trend			<0.0001		<0.0001		0.002	
**CVD**								
Low CRF	112	37.8	1.00	Referent	1.00	Referent	1.00	Referent
Moderate CRF	131	20.1	0.53	0.41–0.69	0.55	0.42–0.72	0.66	0.50–0.87
High CRF	86	14.4	0.38	0.28–0.51	0.40	0.29–0.55	0.55	0.39–0.79
*p* linear trend			<0.0001		<0.0001		0.001	
**Cancer**								
Low CRF	68	22.4	1.00	Referent	1.00	Referent	1.00	Referent
Moderate CRF	96	15.0	0.67	0.49–0.92	0.72	0.52–0.99	0.76	0.54–1.07
High CRF	96	16.6	0.74	0.53–1.03	0.83	0.57–1.20	0.93	0.62–1.40
*p* linear trend			0.13		0.45		0.88	

Abbreviations: HR, hazard ratio; CI, confidence interval; CVD, cardiovascular disease; CRF, cardiorespiratory fitness. * Rate is expressed as per 10,000 person-years and adjusted for age and examination year. ^†^ Adjusted for age and baseline examination year. ^‡^ Adjusted for the above plus baseline total cholesterol (mg/dL), current smoking (yes or no), heavy alcohol intake (yes or no), physically inactive (yes or no), and parental history of CVD (when all-cause or CVD mortality was the outcome) or cancer (when cancer mortality was the outcome) (present or not). ^§^ Adjusted for the above plus body mass index (kg/m^2^) and presence or absence of hypertension or diabetes.

## Data Availability

The datasets analyzed during the current study are not publicly accessible but are available upon reasonable request from the corresponding author.
